# *getSequenceInfo*: a suite of tools allowing to get genome sequence information from public repositories

**DOI:** 10.1186/s12859-022-04809-5

**Published:** 2022-07-08

**Authors:** Vincent Moco, Damien Cazenave, Maëlle Garnier, Matthieu Pot, Isabel Marcelino, Antoine Talarmin, Stéphanie Guyomard-Rabenirina, Sébastien Breurec, Séverine Ferdinand, Alexis Dereeper, Yann Reynaud, David Couvin

**Affiliations:** 1 Unité Transmission, Réservoir et Diversité des Pathogènes, Institut Pasteur de Guadeloupe, Les Abymes, Guadeloupe, France; 2 Faculté de Médecine Hyacinthe Bastaraud, Université des Antilles, Pointe-à-Pitre, France; 3 Centre d’Investigation Clinique Antilles Guyane, Inserm CIC 1424, Pointe-à-Pitre, France

**Keywords:** Genome sequences, Nucleotide diversity, Assembly, DNA, Repository, Metadata

## Abstract

**Background:**

Biological sequences are increasing rapidly and exponentially worldwide. Nucleotide sequence databases play an important role in providing meaningful genomic information on a variety of biological organisms.

**Results:**

The *getSequenceInfo* software tool allows to access sequence information from various public repositories (GenBank, RefSeq, and the European Nucleotide Archive), and is compatible with different operating systems (Linux, MacOS, and Microsoft Windows) in a programmatic way (command line) or as a graphical user interface. *getSequenceInfo* or gSeqI v1.0 should help users to get some information on queried sequences that could be useful for specific studies (e.g. the country of origin/isolation or the release date of queried sequences). Queries can be made to retrieve sequence data based on a given kingdom and species, or from a given date. This program allows the separation between chromosomes and plasmids (or other genetic elements/components) by arranging each component in a given folder. Some basic statistics are also performed by the program (such as the calculation of GC content for queried assemblies). An empirically designed nucleotide ratio is calculated using nucleotide information in order to tentatively provide a “NucleScore” for studied genome assemblies. Besides the main gSeqI tool, other additional tools have been developed to perform various tasks related to sequence analysis.

**Conclusion:**

The aim of this study is to democratize the use of public repositories in programmatic ways, and to facilitate sequence data analysis in a pedagogical perspective. Output results are available in FASTA, FASTQ, Excel/TSV or HTML formats. The program is freely available at: https://github.com/karubiotools/getSequenceInfo. getSequenceInfo and supplementary tools are partly available through the recently released Galaxy KaruBioNet platform (http://calamar.univ-ag.fr/c3i/galaxy_karubionet.html).

**Supplementary Information:**

The online version contains supplementary material available at 10.1186/s12859-022-04809-5.

## Background

Sequencing technologies are widely used nowadays and sequencing data is increasing at a rapid rate. Whole genome sequencing (WGS) projects can be applied in various settings and scientific studies, fostering the analysis of a large amount of data which are generally deposited in public archives such as those belonging to the International Nucleotide Sequence Database Collaboration (INSDC), which comprises the DNA Data Bank of Japan (DDBJ), the European Nucleotide Archive (ENA), and GenBank [1–4]. Unlike GenBank sequences, RefSeq sequences are not part of the INSDC but are derived from INSDC sequences to provide non-redundant curated data representing our current knowledge of known genes. Differences between GenBank and RefSeq genome assemblies are well described (https://www.ncbi.nlm.nih.gov/books/NBK50679/#RefSeqFAQ.what_is_the_difference_between_1).

Several important and useful initiatives have been conducted to foster data retrieval from these public sequence repositories, and various programs or online tools are available for this task. Among these programs/tools, we can non-exhaustively mention the NCBI Genome Downloading Scripts (https://github.com/kblin/ncbi-genome-download), Kraken_db_install_scripts (https://github.com/mw55309/Kraken_db_install_scripts), Entrez Programming Utilities (https://www.ncbi.nlm.nih.gov/books/NBK25501/; https://www.ncbi.nlm.nih.gov/books/NBK179288/), enaBrowserTools (https://github.com/enasequence/enaBrowserTools), NCBI genomes FTP site (https://ftp.ncbi.nih.gov/genomes/), and the recently released NCBI Datasets (https://www.ncbi.nlm.nih.gov/datasets/). Another interesting software tool named metatools_ncbi (https://github.com/farhat-lab/metatools_ncbi) allows downloading Biosample and SRA run information metadata from the NCBI. SRA tools such as fastq-dump (https://github.com/ncbi/sra-tools) or SRAdb [5] are also interesting for downloading FASTQ reads and associated information. Furthermore, a wide range of additional bioinformatics resources are available in the INSDC and elsewhere, adhering to the Findable, Accessible, Interoperable, Reusable (FAIR) data principles, which are fostering better practices in sequencing data analyses [6]. Some research studies are becoming more and more complex due to a wide variety of biological data and formats. Sharing information in a more reproducible, understandable and pedagogical manner is important to make scientific research affordable and accessible to a greater number of scientists and/or students.

Dedicated strategies have been developed to better organize and analyze genomic data such as the notion of genome assembly [7]. A wide variety of software tools have been developed to tentatively respond to specific or more general requests regarding sequence analysis. Nucleotide sequence data could be more or less complex to study, depending on the species of interest or the studied genomic elements/components (e.g. chromosomes, plasmids, etc.). Approaches and methods facilitating comparative genomic studies have been implemented such as the well-known average nucleotide identity (ANI) which allows improving taxonomic assignments [8].

Despite the usefulness of public genomic repositories, metadata related to genomic sequences are not always well annotated or available. As mentioned before [9], the World Health Organization (WHO) has repeatedly advocated open sharing of pathogen genetic sequences as well as the knowledge and benefits resulting from the genetic data (https://www.who.int/blueprint/meetings-events/meeting-report-pathogen-genetic-sequence-data-sharing.pdf). We aim to develop intuitive software to obtain genomic sequences and their associated data when available in a relatively simple way through a graphical user interface (GUI) or using the command line. Such an approach could be of relevant significance for the design of specific biological databases or the improvement of existing ones.

## Implementation and results

### Programming language and modules

The Perl programming language was used to develop the freely available getSequenceInfo (gSeqI) software. Perl/Tk graphical user interface toolkit has been employed to design the GUI in order to make an intuitive interface. Several other Perl modules are required to run the software, including BioPerl (http://bioperl.org/) [10]. The list of needed modules is provided in the software installation file (https://github.com/dcouvin/getSequenceInfo/tree/master/install). Further details regarding programming are available on the software GitHub page (https://github.com/karubiotools/getSequenceInfo). A user manual is also available from the GitHub page to help using the tool. The software is compatible with Linux and Microsoft Windows operating systems.

### How getSequenceInfo tool differs from other existing tools

Unlike other existing tools, getSequenceInfo allows users to query specific information regarding wanted sequences. For example, users can download NCBI sequences from a given release date, and they can also download specific sequences (from the assemblies) such as plasmids, chromosomes, etc. Users can query both ENA and NCBI GenBank or RefSeq databases. Extraction of metadata associated to genome assemblies such as country, host, or isolation source is.

Furthermore, one may notice the notion of “NucleScore” (further explanations are provided below) which could bring new information for classification/characterization of genome assemblies. This score is a reduction of the nucleotide information which is intended to be discriminating and informative. To calculate the NucleScore, measures such as nucleotide variance, GC content, genome size, and AT/GC ratio were used. It allows different species to be distinguished on the basis of nucleotides alone (the use of a reference genome is not necessary).

### Main options

Users can select two methods for querying nucleotide databases managed by the gSeqI software: (i) the method based on NCBI databases (GenBank and RefSeq) designed to download FASTA and GenBank files associated to various metadata (when available), or (ii) the method based on ENA designed to download particularly FASTQ files (among others). Both methods provide different functionalities allowing users to download various sequence file formats. The tool provides a parser allowing to extract assembly metadata (such as country, PubMed ID, isolation source, host, etc.) from FASTA and GenBank files. Specific string search and regular expressions were used to retrieve metadata (further details are provided in Supplementary_tools scripts “nucleScore.pl” and “genbank_info.pl”). Some options allow users to determine several features of their search. Users can select the sequence database from which they want to download genomic data. For example, users can choose a NCBI sequence database with the option “-directory” or “-dir” followed by the database of interest (i.e. “genbank” or “refseq”). Once the database has been selected, the user can indicate the species with option “-species” followed by the wanted species (e.g. *Escherichia coli*). Users can also query the sequence database by indicating a NCBI Taxonomy ID (Taxid) with the option “-taxid”. Furthermore, users can limit the number of NCBI assemblies they want to download (with the option “-n”). Regarding options dedicated to ENA sequence database, users can choose the option “-ena” to download FASTA sequence records from ENA according to an accession number (https://ena-docs.readthedocs.io/en/latest/retrieval/general-guide/data-classes.html) or the option “-fastq” to download compressed FASTQ files obtained from the ENA run accession numbers (starting with “ERR…” or “SRR…”) provided (https://ena-docs.readthedocs.io/en/latest/submit/general-guide/accessions.html). Further options are available for both querying methods (i.e. based on NCBI databases or on ENA) and are visible in the provided user manual (https://github.com/karubiotools/getSequenceInfo/blob/master/User_Manual.pdf).

### Metrics and NucleScore calculation

Common and customized genome metrics (GC content, AT/GC ratio, nuc%, and variance of nuc%) were used to calculate the NucleScore in order to potentially assess genome assembly quality and completeness.

The GC content is calculated as follows: $GC \;content = \frac{G + C}{{A + T + C + G}} \times 100$.

AT/GC ratio is calculated as follows: $AT/GC \;ratio = \frac{A + T}{{G + C}}$.

The percentage for each nucleotide (nuc%) is calculated as follows:$nuc\% = \frac{nuc}{{A + T + C + G}} \times 100$.

The variance (Var) was calculated taking into account the nuc% value for each nucleotide using the basic formula (https://fr.wikipedia.org/wiki/Variance_(math%C3%A9matiques)).

The NucleScore was calculated taking into account the variance Var, the GC content, the AT/GC ratio, and the total sequence length (using log2 and square root to tentatively normalize the result) as follows:

$$ Nucle\;Score = log_{2} \left( {\frac{{Var \times GC\;content \times ATG\;Cratio^{3} }}{{\sqrt {length} }}} \right) $$

Length variable represents the total size of a given genome assembly.

**Fig. 1:**
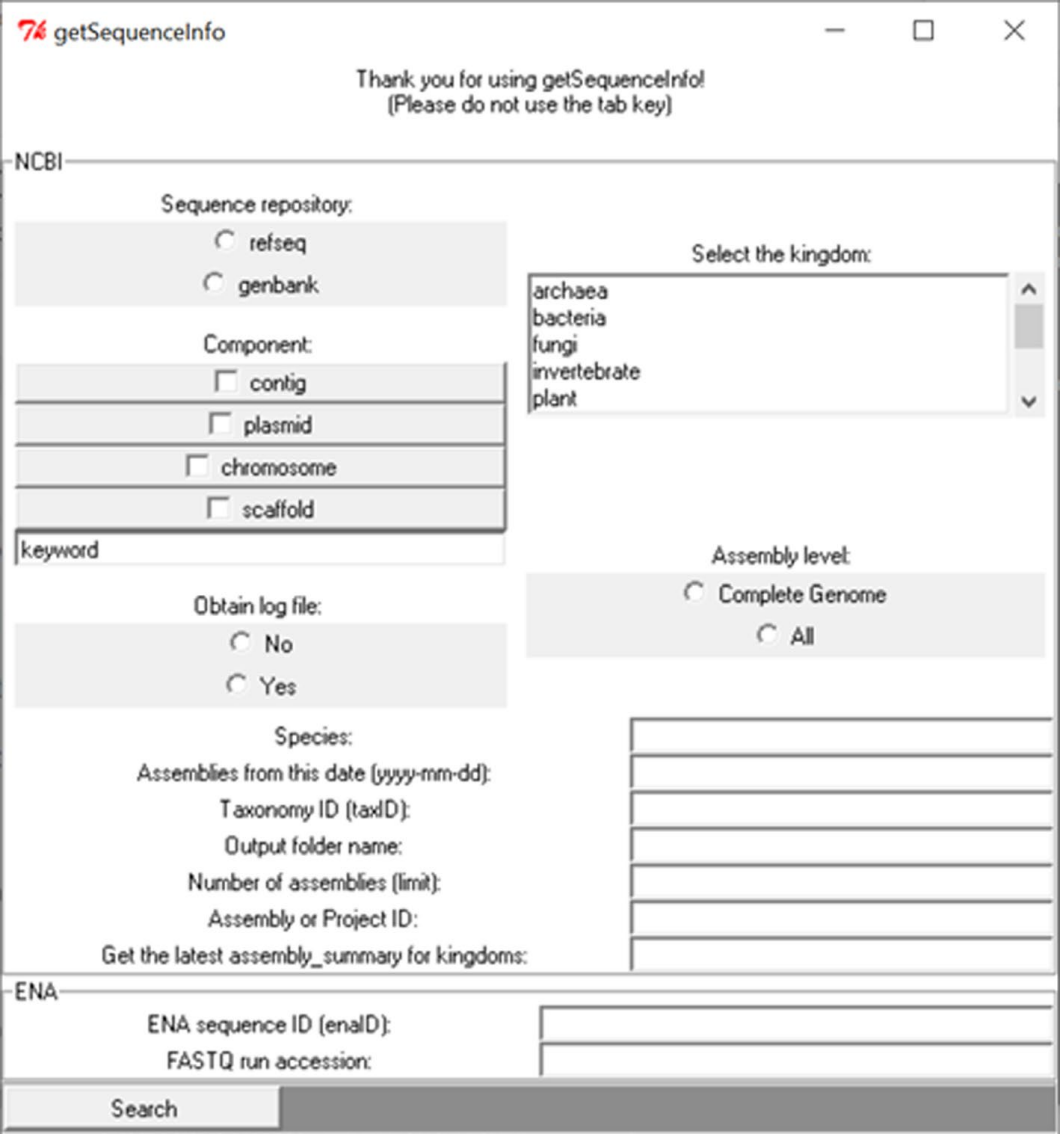
Overview of the GUI of getSequenceInfo

A graphical user interface (GUI) has been developed to facilitate the utilization of the program (Fig. 1).

### Examples of use

Note that the ‘$’ symbol indicates a shell prompt (or command terminal).

(i) Coronavirus genome assemblies (example of 50 genomes available from December 1st 2019)
  $ perl getSequenceInfo.pl -s coronavirus -k viral -date 2019-12-01 -n 50 -o COVID19.
  Please note that nowadays, a wide range of specific tools have been developed for querying SARS-CoV-2 data such as this NCBI resources page: https://www.ncbi.nlm.nih.gov/sars-cov-2/.
(ii) *E. coli* complete genome assemblies from RefSeq and their associated plasmids (example of 50 genomes)
  $ perl getSequenceInfo.pl -k bacteria -s “Escherichia coli” -d refseq -level “Complete Genome” -c plasmid -n 50.
(iii) 5 sequences belonging to *Naegleria fowleri* species from GenBank
  $ perl getSequenceInfo.pl -k protozoa -s “Naegleria fowleri” -d genbank -n 5.
(iv) FASTA sequences and associated text reports from ENA
  $ perl getSequenceInfo.pl -ena GCA_000195955,BN000065.
(v) FASTQ run accessions from ENA (example with four Brazilian *M. bovis* genomes)
  $ perl getSequenceInfo.pl -fastq SRR7693912,SRR7693877,SRR9850824,SRR9850830.

### Supplementary tools

A set of additional or complementary tools is also offered to help users analyze their DNA sequences.

These tools are available in a dedicated folder named “Supplementary_tools” on the GitHub repository.

A dedicated program named “nucleScore.pl” was designed to get metrics corresponding to the aforementioned “NucleScore” directly from users’ data. The code can be used as follows (considering a set of FASTA files available in the current repository):

$ perl nucleScore.pl *.fasta.

The result file will be a tabulated file containing sequence information corresponding to each FASTA file (nucleotide frequencies, GC-content, AT/GC ratio, etc.).

Another program named “countDifferences.pl” allows users to compare sequences from a multi-Fasta alignment file by calculating differences (in bp) and percentage identity. The code is used as follows:

$ perl countDifferences.pl multi_alignment.fasta.

It generates percentage_identity and distance matrices from the given multi-FASTA alignment input file.

Then, a program named “SRArunInfo.pl” allows users to get information on running accessions using ID (e.g. SRR7693912). The program can be runned as follows:

$ perl SRArunInfo.pl SRR7693877,SRR9850824,SRR9850830.

A result summary information corresponding to the query is generated (Additional file 1). In addition, XML and CSV result files are generated for each queried accession.

“removeChar.pl” tool allows to remove positions (or columns) from a multi-Fasta alignment file in function of a given character (e.g. ‘N’ or ‘-’). The program can be used as follows:

$ perl removeChar.pl alignment.fasta -

The result will be a multi-Fasta alignment file without the given character (‘-’) in the sequences.

### Availability through the Galaxy KaruBioNet instance

**Fig. 2:**
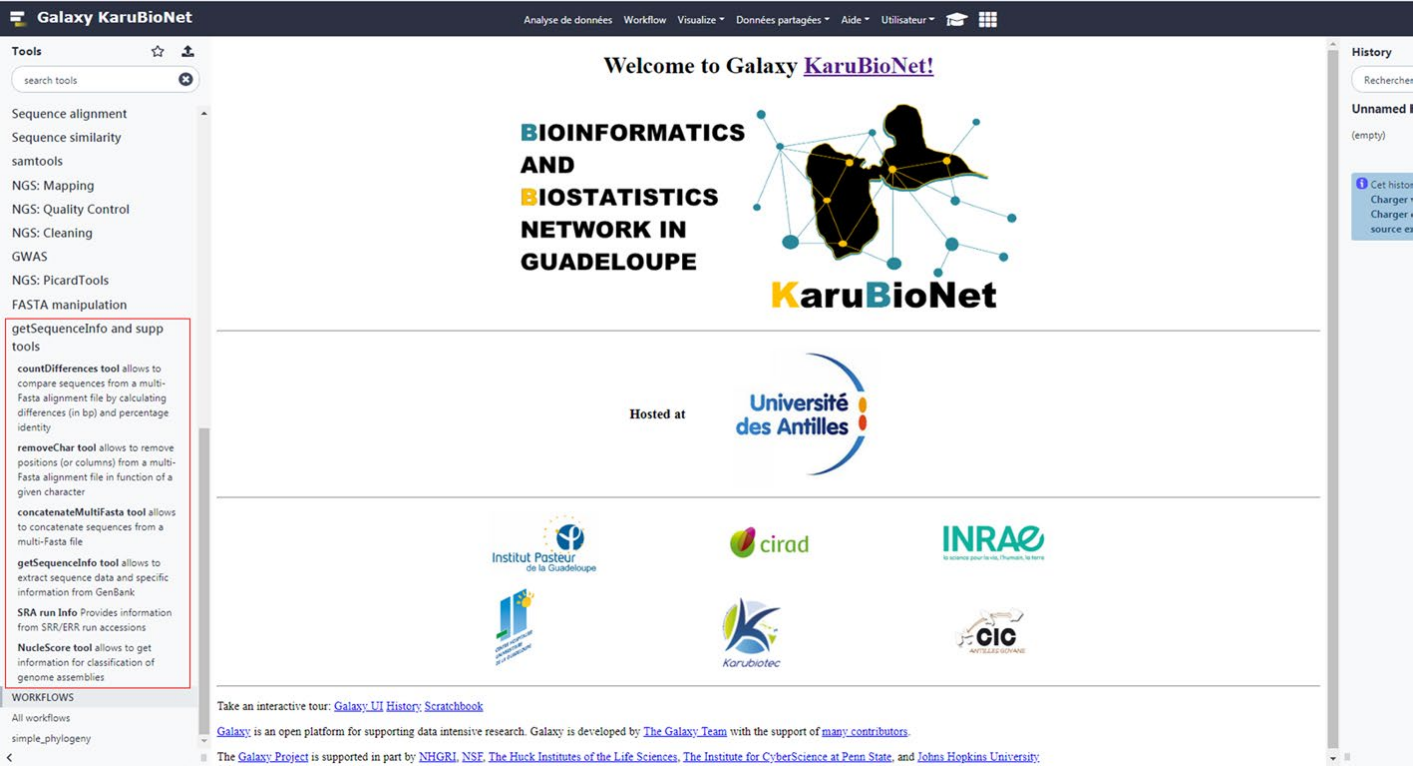
Galaxy KaruBioNet screenshot with gSeqI and additional tools framed in red

Partial functionalities of the getSequenceInfo and supplementary tools have been made available through the Galaxy KaruBioNet instance (http://calamar.univ-ag.fr/c3i/galaxy_karubionet.html) [11]. Users who are not comfortable with the command line interface (CLI) or the GUI can use Galaxy [12] to perform the analysis in an even more user-friendly way. Furthermore users can easily register and login to the website providing an email address and a password. A screenshot of the welcome page of the Galaxy KaruBioNet highlighting the tool suite is shown in Fig. 2. Another tool named catchSequenceInfo (available in our Galaxy instance) allows to get resistance, virulence, plasmids, and multilocus sequence typing (MLST) [13] information from 2,518 complete genome assemblies (mainly collected from RefSeq). This tool uses: (a) ABRicate (https://github.com/tseemann/abricate) with ResFinder [14], PlasmidFinder [15], and VFDB [16] databases to predict resistance, plasmid, and virulence genes; as well as (b) MLST tool (https://github.com/tseemann/mlst). A percentage coverage of 90.00% was used to screen resistance and plasmid genes, whereas a percentage coverage of 80.00% was used to filter virulence genes. getSeqI and catchSequenceInfo tools were used to build a small dataset containing assemblies belonging to *Escherichia coli* (n = 1835), *Klebsiella pneumoniae* (n = 622) and *Enterobacter cloacae* (n = 61) species (results are provided in Additional file 2). Please note that due to our low data storage capacity, the total number of assemblies that can be downloaded has been limited to 50 in Galaxy (users can use the CLI interface to download further data). Note that, after using getSequenceInfo tools from Galaxy, users are invited to delete and purge generated data.

### Potential use of NucleScore for bacterial organisms delineation

NucleScore calculation was tentatively used to potentially delineate bacterial organisms.

**Fig. 3:**
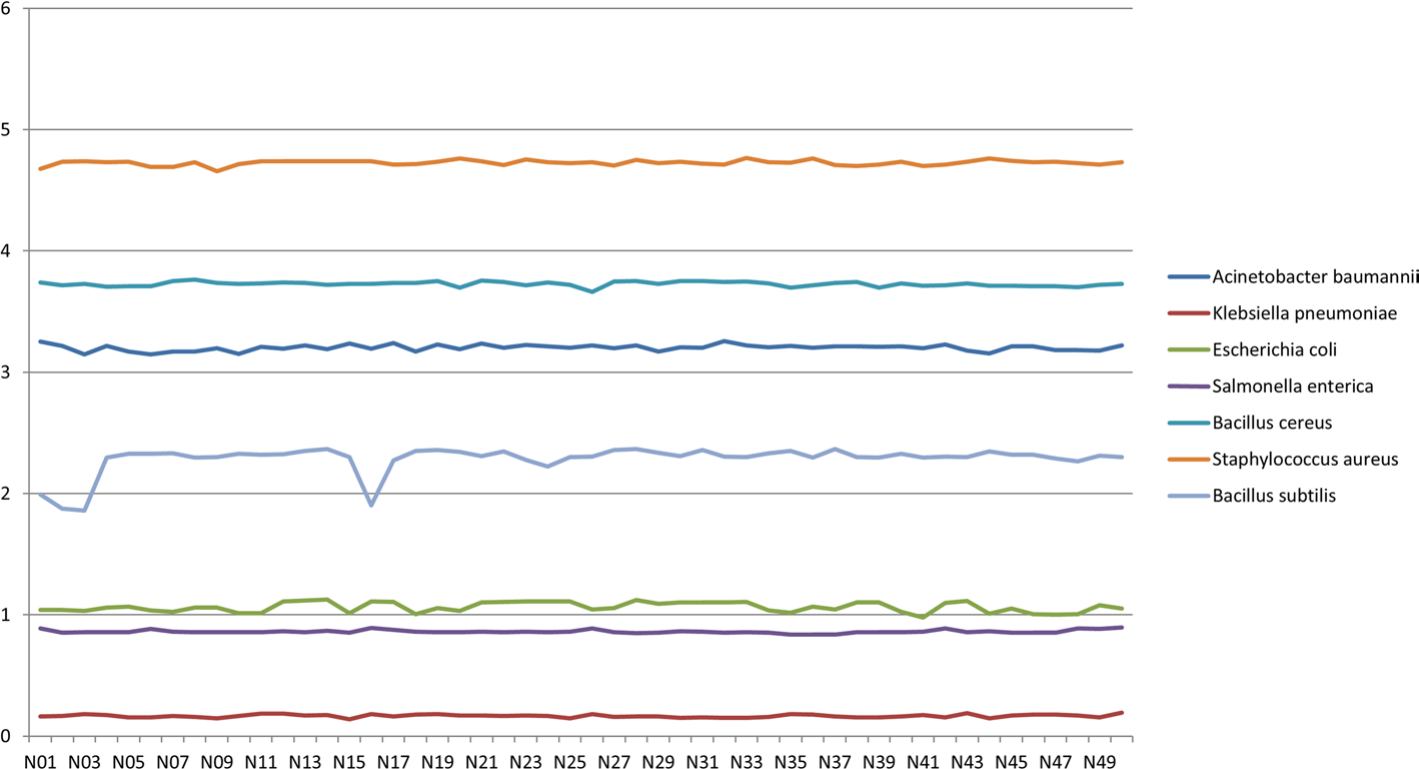
NucleScore value of 50 genomes belonging to 7 different bacterial species (*Acinetobacter baumannii*, *Bacillus cereus*, *Bacillus subtilis*, *Escherichia coli*, *Klebsiella pneumoniae*, *Salmonella enterica*, *Staphylococcus aureus*)

NucleScore values varied by species as follows (see Fig. 3):

(i) *Staphylococcus aureus* (min = 4.655; max = 4.768)
(ii) *Escherichia coli* (min = 0.978; max = 1.125)
(iii) *Acinetobacter baumannii* (min = 3.146; max = 3.256)
(iv) *Salmonella enterica* (min = 0.838; max = 0.895)
(v) *Bacillus cereus* (min = 3.66; max = 3.764)
(vi) *Bacillus subtilis* (min = 1.858; max = 2.369)
(vii) *Klebsiella pneumoniae* (min = 0.140; max = 0.195)

The NucleScore metric can potentially be used as a score to assess the quality or completeness of a genome assembly.

We can notice that some species (such as *Staphylococcus aureus* and *Klebsiella pneumoniae*) are easily identifiable thanks to their NucleScore. However, other species may be difficult to identify based on their NucleScore (e.g. scores of *Escherichia coli* and *Salmonella enterica* are very close). This may constitute a limitation to the use of the NucleScore.

## Discussion

The getSequenceInfo software tool attempts to allow users to easily download sequences as well as associated metadata from NCBI’s GenBank or RefSeq and EBI’s ENA without advanced computing knowledge or complex manipulations.

This tool has been designed to work preferably on a small number of sequences of interest. It could be helpful in designing specific genomic databases or strains collection, by facilitating access to sequences using accession numbers and keywords. For example, users can easily download plasmid sequences from available strains belonging to given species (e.g. *Escherichia coli*).

Although existing sequence downloading tools offer great functionality to download sequences in various manners, getSeqI provides a different way to download sequences making association with several metadata (such as country and host), and allowing users to make specific queries.

The main tool has also been made accessible through a Singularity container to facilitate its utilization (the GitHub page of the tool shows how to install the Singularity image) [17]. The tool will also be deposited in a Docker container (https://docs.docker.com/). Prospects for improvement of this tool will be performed. The fact that the software tool is for the moment available as a standalone program (although it is also available through a GUI) could represent a limit for the utilization of the tool. Therefore, efforts have been made to make the getSequenceInfo tool partly accessible through a Galaxy platform. Future developments of getSequenceInfo will consist in adding some functions for improving extraction of useful information and in adding other methods to make the tool more accessible. Moreover, additional programming tools (using machine learning and other techniques) will be applied to improve the developed tools and enhance the prediction/calculation of the NucleScore. Several methods exist for the delineation of bacterial genomes [18], and we can draw inspiration from them to improve our methodology. For now, the getSeqI tool can be used to download a small amount of sequence data (< hundreds). The ability to clearly define sequences of interest and the number of sequences that can be downloaded represent the limits of this tool. However, despite the limits concerning the NucleScore, it nevertheless makes it possible to identify certain species in a fairly simple way. Furthermore, we believe that future investigations and improvements will potentially make this score more relevant.

Some initiatives such as the Nagoya Protocol on Access and Benefit Sharing (https://en.wikipedia.org/wiki/Nagoya_Protocol) and the Convention on Biological Diversity (https://en.wikipedia.org/wiki/Convention_on_Biological_Diversity) could play a role in helping nations for a better managing of biological resources. We promote a fair sharing of bioinformatics resources. Strategies are also needed to make scientific results more accessible and affordable for all interested people (https://en.wikipedia.org/wiki/Open_science). In our opinion, it is important to facilitate the understanding of biological sequence analysis for a large audience by making/sharing less complex, simpler, low-cost, reproducible and accessible methodologies.

## Conclusion

To conclude, getSequenceInfo offers an accessible way to get genome sequences (in FASTA, FASTQ or GenBank formats) and associated metadata in a programmatic way (command line) or using a graphical user interface (GUI). The software tool allows users to get multi-level information from queried genome assembly such as country of origin/isolation, release date, host, etc. (using NCBI GenBank or RefSeq databases). getSequenceInfo could allow users to retrieve sequencing data in the function of a given kingdom or species, and from a given date. The separation between chromosomes and plasmids (or other elements/components) by ranging corresponding sequences in a given folder, is also possible. Some basic statistics are also performed by the program (such as the calculation of GC content for queried assemblies). Furthermore, an empirically designed nucleotide ratio (“NucleScore”) is proposed to tentatively provide a score explaining nucleotide diversity of queried genome assemblies. Supplementary tools are provided to help users with some occasional needs for sequence analysis and data retrieval. getSequenceInfo and tools are partly available from the recently released Galaxy KaruBioNet platform (http://calamar.univ-ag.fr/c3i/galaxy_karubionet.html). Finally, getSequenceInfo could be used as an educational tool allowing students to better comprehend sequence data.

## Availability and requirements

Lists the following:

- Project name: getSequenceInfo
- Project home page: https://github.com/karubiotools/getSequenceInfo
- Operating system(s): Platform independent
- Programming language: Perl
- Other requirements: e.g. Perl 5.26 or higher, Perl modules
- License: e.g. GNU GPLv3.
- Any restrictions to use by non-academics: licence needed

## Supplementary Information

**Additional file 1:** Result dataset obtained using “SRArunInfo.pl” for 3 accessions (SRR7693877, SRR9850824, and SRR9850830).

**Additional file 2:** Result datasets corresponding to 2,518 complete genome assemblies belonging to *E*. *coli*, K. pneumoniae, and E. cloacae from RefSeq or GenBank repositories. The first sheet shows results obtained from gSeqI, the second sheet provides catchSequenceInfo results, and the following sheets provide Sequence Types (ST) distribution for each species.

## Data Availability

All data is available on the GitHub repository (https://github.com/karubiotools/getSequenceInfo). A folder named "datasets" contains additional files that were used in the manuscript as output examples.

## Abbreviations

gSeqI: GetSequenceInfo tool
GUI: Graphical user interface
CLI: Command-line interface
nuc: Nucleotide
GC-content: Guanine-cytosine content
